# Ethical tensions in disaster coverage: a qualitative thematic analysis of rescue-moment broadcasting after the 2023 turkey earthquake

**DOI:** 10.3389/fpubh.2026.1725269

**Published:** 2026-01-23

**Authors:** Ayse Kurtoglu, Abdullah Yildiz, Berna Arda

**Affiliations:** Department of Medical History and Ethics, Ankara University School of Medicine, Ankara, Türkiye

**Keywords:** bioethics, disaster ethics, media ethics, privacy, qualitative study

## Abstract

**Background:**

Disaster coverage informs the public yet may expose victims at moments of extreme vulnerability. This study examined how televised rescue-moment broadcasting after the 6 February 2023 Turkey earthquake intersected with bioethical principles relevant to public health.

**Methods:**

Using reflexive thematic analysis, we analyzed 60 rescue-moment videos from two national broadcasters (public and private). Focusing strictly on presented content, we coded ethically salient visuals and discourses (identity disclosure, medical interventions, and emotional framing) and developed themes through iterative reflection among researchers.

**Results:**

Two overarching categories characterized coverage: (1) normalized discursive presentation—open identity disclosure, emotional dramatization, and routinized live “rescue shows”; and (2) privacy-insensitive visual content—unblurred faces, filming of medical interventions, and overt displays of trauma amid crowd convergence.

**Conclusions:**

These practices often conflicted with privacy, dignity, and non-maleficence, underscoring the need for trauma-informed disaster communication.

**Limitations:**

We analyzed content only, not journalists intentions or newsroom processes.

**Implications:**

Findings inform ethical guidelines for disaster reporting and training programs integrating bioethics and journalism ethics.

## 1 Introduction

Disasters are events that deeply affect both the local population and humanity as a whole from a humanitarian and moral perspective (1). The devastating earthquake that struck several cities in Turkey on February 6, 2023, forced humanity to face many humanitarian and ethical issues again (2, 3). Disasters inevitably create new morally significant circumstances (4). As society grapples with sudden challenges, new ethically vulnerable groups emerge. In disasters, society is affected, from the individual who runs for help to the individual who is rescued. On the one hand, while society's feelings of compassion and charity increase, individuals who were previously part of regular everyday relational networks may find themselves in asymmetrically weakened positions in humanitarian and ethical positions in proportion to the degree to which they are affected by the disaster (5). In parallel, the sudden onset of disasters and the need for emergency responses that deviate from daily routine practices complicate ethical decision-making processes and bring with them the risk of ethical problems (6). Therefore, the complex nature of disasters and their instantaneous occurrence, including many emotional reactions, make it important for each subject (institutional or individual) involved in this process to reflect on the ethical dimension of their actions (7). In this context, it can be said that some groups play extremely important roles in disaster processes. For example, health workers, rescue workers, security forces, and public officials are some of them. Undoubtedly, one of these important subjects is the media and news organizations and their representatives in disaster areas, who are responsible for informing the public about disasters (8, 9).

The earthquake of February 6, 2023, deeply shook Turkey and the world, and a significant part of the country and the world followed the rescue efforts through various media platforms, especially television channels. Television broadcasts, in particular, have become the primary source of information for millions of people and a source of emotional input to the disaster areas. The media plays a crucial role in disseminating information during natural disasters, providing real-time updates to the public, understanding problem areas related to natural disasters, and providing data to the public to take necessary actions. Moreover, the media's relationship with disasters is not limited to the moment of the disaster. The media plays various roles before and after disasters. In this sense, it is important to sensitize society before disasters and contribute to the necessary preparations. It is also believed that the media have important functions in maintaining individual and social wellbeing during and after disasters. Contributing to the behavioral and mental health of individuals and society, as well as to recovery and resilience after disasters, is also an important area of expectation (10). However, it should also be noted that poor reporting practices carry risks, including ethical issues (8). In addition to these, it is possible that media practitioners, as well as disaster victims, may be affected by the traumatic side of disaster moments. All this makes disasters a morally important issue (11, 12).

Especially in disasters, reporting focusing on the “first moment of the disaster” or its immediate surroundings comes to the fore (13). However, given the broad consideration of the far-reaching functions of journalism, the ethical description of the reporting and broadcasting in the first moment provides an important basis for thinking about the meanings of this news that extend beyond the moment of the disaster. In addition, identifying the issues that may be ethically problematic will be necessary for further action plans or for understanding the extent to which existing ethical codes are functional. In this context, it is noteworthy that journalists also consider ethical issues in disaster reporting a vital topic. For example, in addition to fast and accurate reporting, having an understanding that includes respecting the personalities and rights of disaster victims and showing empathy to them is one of the most emphasized issues. It is stated that it is necessary to be careful about issues that may cause disaster survivors to be traumatized in the long process and that confidentiality should not be violated (8, 14).

As some journalists and reporters dealing with disasters state, ethical issues should be constantly on the reporter's mind (8). In this context, it can be said that the approaches of media ethics and bioethics to the autonomy, privacy, and rights of the individual and their discourses on the importance of having ethical awareness overlap significantly (4, 14–16). While the suddenness and unusualness of disasters may make ethical awareness difficult for a moment, it should be kept in mind that this issue may have long-term effects as an important issue. Like many others, we first followed the news and information about the February 6 earthquake through the media, primarily television broadcasts. As bioethicists watching the media coverage of the rescue efforts, we realized that there were some troubling ethical issues in presenting images and news about the rescue moments in these disasters. In particular, we thought there might be some problems with the interventions regarding individuals who could be considered victims at the time of rescue from the earthquake, especially considering the concepts of confidentiality and privacy, which are well-established bioethical principles (17). However, understanding whether this is a judgment based on our emotional intuition in the first moment or whether it is empirically relevant was considered an ethically important evaluation issue (18). Thus, this was the primary motivation for conducting this research.

While medical and humanitarian ethics in disaster response have been widely studied, the ethical dimensions of disaster *media* coverage remain comparatively underexplored. Previous research has shown that journalistic practices during crises can profoundly affect both the dignity and psychological wellbeing of victims and the emotional resilience of journalists themselves (19). While bioethics emphasizes non-maleficence, privacy, and respect for autonomy, disaster journalism introduces further complexities involving the public's right to know, immediacy, and the emotional framing of suffering. International organizations such as the Dart Center for Journalism and Trauma and the Ethical Journalism Network (EJN) advocate trauma-aware standards—such as harm minimization, humanity, and accountability—to support ethically responsible journalism under extreme conditions. Similarly, Ewart and McLean (20) outline and emphasize the possibility of best practice approaches that balance timeliness, accuracy, and empathy in disaster reporting. These frameworks remind us that ethical disaster journalism requires sensitivity to both victims vulnerability and journalists exposure to trauma. More recently, Mishra (21) proposed codifying disaster journalism ethics as an emerging subfield, emphasizing context-sensitive adaptation of global principles. Together, these works frame our study within a broader interdisciplinary context where bioethics, media ethics, and public health ethics intersect. This study contributes to that dialogue by analyzing televised rescue coverage of the 2023 Turkey earthquake through a qualitative thematic analysis, identifying how ethical tensions between public information and individual dignity manifest in real-time broadcasting.

## 2 Methods

### 2.1 Research design

This study employed qualitative research using reflexive thematic analysis to examine the ethical dimensions of media coverage during rescue operations following the February 2023 Turkish earthquake. This approach was chosen for its capacity to identify and analyze patterns of meaning within complex social phenomena, particularly in ethically sensitive contexts, while acknowledging the active role of researchers in the interpretive process (22, 23). Our research design emphasized a descriptive yet interpretive orientation, allowing for systematic analysis of ethical implications in disaster coverage. Our analysis targets publicly broadcast content and its ethical implications. We do not evaluate journalistic intent, newsroom decision-making, or causal audience effects. Reflexive thematic analysis is suited to identifying patterns of ethical meaning in representations without assuming objectivist reliability or triangulation.

### 2.2 Data collection and processing

The dataset comprised rescue moment coverage from two major Turkish broadcasters (one public, one private) during the immediate aftermath of the February 6, 2023 earthquake. In total, 60 videos (30 from each broadcaster) were analyzed, including television broadcasts and associated YouTube content. During our analysis, we observed that these broadcasters and other national television channels, frequently utilized content from the same press agencies, suggesting that our sample captured patterns representative of broader national media coverage during the disaster. Initial analysis of 20 videos from each broadcaster revealed significant information power and similar patterns, but sampling continued until the final documented rescues to ensure comprehensive temporal coverage. Two bioethics experts (AK and AY) watched all videos simultaneously and collaboratively transformed the visual and verbal content into a written format for analysis. This collaborative viewing approach enhanced the depth of ethical analysis while maintaining consistency in interpretation. Data processing included recording temporal markers (days post-earthquake), broadcast identification, video reference/link, and content description, including ethically significant visual and verbal content.

### 2.3 Analysis

The analysis was conducted collaboratively by AK and AY using MAXQDA software (version 2022, VERBI Software, Berlin, Germany), following Braun and Clarke's reflexive thematic analysis approach. Rather than seeking mechanical coding agreement, our analysis emphasized organic theme development through researcher engagement and dialogue (22, 24). The analysis proceeded through several phases:

Familiarization: Both researchers viewed all videos together, discussing and documenting initial observations about ethical implications.
Data transformation: Collaborative verbalization of video content into written format, with particular attention to ethically significant moments.
Coding process: Systematic coding of ethically significant content using MAXQDA.
Theme development: Collaborative identification of emerging patterns through regular discussion and reflection.
Theme refinement: Joint review and definition of themes, emphasizing ethical dimensions.
Final analysis: Integration of themes with theoretical frameworks from bioethics and media ethics.

This collaborative approach allowed for rich dialogue between researchers and deeper engagement with the ethical implications of the content, aligning with what O'Connor and Joffe (25) describe as enhancing analysis through multiple perspectives rather than seeking strict coding consensus.

### 2.4 Researcher positionality and research quality

As bioethicists analyzing media content, we acknowledge the influence of our theoretical and professional backgrounds on our analysis. Our bioethical training inevitably shaped our attention to human dignity and vulnerability issues. However, we maintained a primarily descriptive orientation while allowing our bioethical expertise to inform our analytical sensitivity (26). This position enabled us to identify ethically significant patterns while remaining open to emergent themes in the data. To ensure research quality and trustworthiness, we systematically documented analytical decisions and maintained regular collaborative viewing and analysis sessions. The collaborative nature of our analysis, involving two researchers with bioethics expertise, facilitated rich dialogue and cross-checking of interpretations. Throughout the process, we maintained detailed documentation of ethically significant content and ensured transparent linking of our findings to empirical evidence. These strategies align with established criteria for qualitative research trustworthiness (27) while maintaining sensitivity to the ethical dimensions of disaster coverage. Our approach to ensuring rigor was thus intrinsically connected to our positioning as bioethicists, allowing us to leverage our expertise while maintaining critical awareness of its influence on our analytical process.

### 2.5 Ethical considerations

Given the sensitive nature of disaster coverage and the vulnerability of affected individuals, particular attention was paid to maintaining respect for human dignity and privacy throughout the analysis. While all analyzed content was publicly available, the research team approached the material with heightened ethical awareness, particularly regarding the presentation of findings. This ethical orientation influenced both our analytical process and the way findings were presented.

## 3 Results

The broadcasts analyzed revealed limited visible attention to privacy or dignity safeguards during live or recorded rescue coverage. Our analysis does not assign fault or intent but focuses on content features that raise ethical concerns about how bioethical principles are enacted—or absent—within disaster journalism. This approach distinguishes between ethical concern and ethical violation, emphasizing descriptive, not accusatory, interpretation. While the study primarily examined visual material, ethically significant discursive elements also emerged and were included in the dataset (27). We use the term “raises an ethical concern” to describe content features that plausibly conflict with privacy, dignity, autonomy, or non-maleficence, without implying misconduct or intent. “Normalized discursive presentation” refers to routinized broadcasting patterns—naming, dramatization, and repetitive live “rescue shows”—that make ethically sensitive depictions appear ordinary. “Spectacularization” denotes stylistic amplification of emotion that prioritizes dramatic effect over privacy protection. Thus, the analysis revealed two main categories of ethical concerns: Normalized Discursive Presentation and Privacy-Insensitive Visual Content. These categories and their corresponding themes are summarized in Table 1. Because the analysis was based on media content, content emerged where the descriptive dimension was more evident. The following sections present these categories in detail, illustrating how ethical tensions materialized in language, imagery, and broadcasting routines.

**Table 1:** Ethical concerns in disaster journalism: categories, themes, and representative examples from the Turkey earthquake 2023.

**Category**	**Themes**	**Description**	**Representative contents or examples**
Normalized discursive presentation	Open disclosure of individual identity	Routine disclosure of victims' personal details such as names, ages, and other identifying information.	When the focus returns to the wreckage, the announcer gives the name and details of the individual and the moment of rescue is again in focus (without any masking or blurring). The broadcast lasts about 10 min and includes many details about the victim. (B-1/I-18)
	Emotional and dramatic framing	Use of emotionally charged language and dramatic visuals in disaster reporting. The use of terms such as “miracle” and “tearful reunion” in the broadcast.	Meanwhile, it can be seen that information is being given about the mother that can be considered private. The paramedic gives the information as “25 years old, 2.5 months pregnant mother” and this information is taken and reported by the announcer. Then a firefighter continues to give information to the press; “The mother was breastfeeding!” he says, and attempts are made to take footage from inside the ambulance. (B-2/I-22) The announcer at the centre of the live broadcast says: “a miracle is happening… in fact, for 3 days we have been sharing countless miracles with you, bringing them to the screens… here he is with his little eyes and his look of wonder (the rescued child is projected on the screen without blurring).” (B-1/I-21)
	Normalized and planned coverage	Coverage that appears to be influenced or directed by interaction with rescue teams. Some rescue workers are showing reporters where the plane wreckage is so that they can film the rescue scenes.	During the long broadcast, clips of previous rescues are shown. Then a screen shows an image of a newly rescued girl being carried on a stretcher to the ambulance. Images of all those rescued are then projected on the screen in the same way. Some rescuers lead reporters into the rubble to film moments of the rescue. (B-1/I-22)
Privacy-insensitive visual content	Open display of rescued individuals	Filming and broadcasting without blurring or masking vulnerable people during rescue operations. Scenes showing injured or rescued people, including children, without blurring.	The face of the child, still under the rubble, is reflected on the cameras. The child is given water. The child is filmed trapped under the rubble and is sometimes seen crying. (B-2/I-14)
	Open broadcast of medical interventions	Recording and broadcasting of medical interventions in the first moments of rescue, including interventions in an ambulance or on a stretcher, without privacy concerns. None of the scenes focusing on moments of medical intervention were blurred or masked.	The moment a child is rescued and brought to the ambulance is shown on the screen. The filming continues inside the ambulance, showing the face of the injured person and the medical examination. There is a dense crowd. The presenter moves the microphone closer to the area to hear what is being said in the ambulance. (Broadcaster-1/Image-6)
	Open presentation of the physical and psychological trauma	Showing visible wounds and psychological distress, including crying and moaning, when broadcasting.	At the beginning of the video, the girl is seen on a stretcher with half her body under the rubble and her face completely open, she is asked her name, she answers, this content is seen completely uncensored. When she is pulled out of the rubble, she is conscious and appears to be in pain. She says “my legs hurt.” (B-2/I-8)
	Rescue under confluence	The presence of large crowds and intense noise (sometimes including religious or patriotic slogans) at rescue sites.	Those who were taken out were carried on a stretcher through a very dense crowd. Many cameras and people were there to record the moment on their mobile phones. The face of the first person rescued cannot be seen from the crowd, but the filming continues. It can be seen that the medical teams have warned to reduce the crowd for the patient's health. (B-1/I-8)

### 3.1 Normalized discursive presentation

It was found that the content of the presentations made by two different media was not significantly different. Moreover, the forms of presentation reflect quite similar content patterns. This category shows an ethically normalized presumption in which principles such as respect for autonomy, which are important parameters of the established ethical discourse, are not considered. The themes under this category are open disclosure of individual identity, emotional and dramatic framing, and normalized and planned reporting.

#### 3.1.1 Open disclosure of individual identity

In a significant portion of the rescue broadcasts, the people's identities, their ages, etc., were clearly presented. From time to time, this content was also visually reflected on the screens. In addition, this content is also included in digital media platforms where live broadcast content is presented. If there is additional information about the individuals, it can be seen that media organs also present this information without hesitation. The impression is that the information content that is not given is related to a lack of access to information rather than ethical sensitivity.

#### 3.1.2 Emotional and dramatic framing

It was found that the discourses accompanying the presentations, made without censorship or softening of the visual content, were generally emotionally stimulating. In this regard, terms such as “miracle” and “tearful reunion” stood out. These narratives often accompanied footage taken from beneath the rubble during rescue operations or visuals of medical interventions. For instance, images of a rescued child speaking with a reporter while still trapped under the debris were presented in an intensely dramatized manner on the television channel:

“*…his frail, fragile body was saved, its tough not to be touched [tearfully]”* (Broadcaster-1/Image-4)

Another rescue moment is depicted with the information that a mother saved her baby by breastfeeding him under the rubble. Their rescue from the debris is presented without any visual obstructions, accompanied by the following statements:

“*… a miracle is happening… in fact, for three days we have been sharing countless miracles with you, bringing them to our screens… here she is, with her little eyes and astonished gaze”* (Broadcaster-1/Image-21)

The following is information about a woman who was rescued from the rubble and her child who was found dead. This content may have a negative impact on the woman's future psychological state. The news link reflecting the aftermath of the earthquake is still available at the time of submitting this article.

“*He told us that he contacted the wounded person, she asked for water, and the first thing she asked as soon as she opened her eyes was about her child, but unfortunately, her child died, and she was transferred by ambulance helicopter.”* (Broadcaster-2/Image-30)

#### 3.1.3 Normalized and planned coverage

It can be observed that live broadcasts and the instantaneous presentation of rescue moments from the rubble are normalized, without critical reflection. Notably, earthquake rescue broadcasts follow a normalized format that is widely accepted without question. Although our analysis focused on two broadcast channels, reporters from various other networks were frequently visible within these broadcasts, occasionally engaging in sidebar reporting.

Over time, it became clear that the live coverage of rescue moments was becoming a distinct broadcast technique. In some cases, there was even an exchange of information between certain rescue teams and reporters about where rescues would occur. In this context, journalists at the rescue sites reported these moments through live connections, turning them into real-time news stories.

This finding suggests that this way of presenting news has become a journalistic ethos or a show. This phenomenon seems related to the visual data showing chaotic scenes at the entrances to the rescue sites. In addition, during the data analysis, verbal expressions associated with these chaotic moments were occasionally observed, as shown in the following excerpts.

“*Some rescue workers direct reporters to the rubble to film the rescue moments.”* (Broadcaster-1/Image-22)

### 3.2 Privacy-insensitive visual content

When the visual content is analyzed from an ethical perspective, it becomes clear that its primary determinant is inconsistent with established ethical discourses on privacy. This suggests that privacy is a central theme within the visual content category. Within this category, three key themes emerge: the open display of rescued individuals, the open broadcast of medical interventions, and the open presentation of the physical and psychological trauma. These findings indicate that the most vulnerable moments of almost all rescued individuals were publicly presented without any safeguards.

Another prominent visual theme is the overwhelming presence of people during rescue operations, as reflected in the footage. This intense confluence raises ethical privacy concerns, further complicating the already sensitive nature of the rescue process.

#### 3.2.1 Open display of rescued individuals

During the rescue, the faces of nearly everyone who was saved and captured on camera were prominently displayed. Notably, cameramen made considerable efforts to film these moments. Individual recording was not limited to national broadcasters; in many instances, phone footage of various people—some of whom were rescue workers—was also aired during national broadcasts. In certain cases, content was directly sourced from third-party mobile devices and presented on screen.

#### 3.2.2 Open broadcast of medical interventions

Aligned with the theme of the open display of rescued individuals, media broadcasts also captured moments of medical intervention and presented them without blurring or censorship. In some footage, cameras even entered ambulances, capturing paramedics performing medical procedures or conducting examinations and anamnesis. Although, in rare cases, health workers attempted to prevent overcrowding and filming, the overall data suggest that their efforts were insufficient primarily to act as a barrier to recording. Given the chaotic environment, expecting them to have the necessary resources to enforce such restrictions effectively also seemed unrealistic.

#### 3.2.3 Open presentation of physical and psychological trauma

In many images, the physical wounds and emotional distress of those rescued from the rubble are visibly displayed on screen. For instance, in one scene, a young girl is shown lying on a stretcher, partially trapped under the debris, with her face and upper body fully exposed. She is asked her name, to which she responds, and this exchange is broadcast without any censorship. She appears conscious and visibly in pain as she is pulled from the rubble:

“*My legs hurt”* (Broadcaster-2/Image-8)

All of this content is broadcast on screens, and it is possible to say that both physical and psychological trauma are clearly visible in these broadcasts. Again, within this theme, content with the potential to cause psychological trauma is occasionally presented through visuals. This aligns with the theme of “emotional and dramatic framing” within the broader discourse on content.

“*He told us that he contacted the wounded person, she asked for water, and the first thing she asked as soon as she opened her eyes was about her child, but unfortunately, her child died, and she was transferred by ambulance helicopter.”* (Broadcaster-2/Image-30)

While this narrative content was being used, the moment when the female patient was transferred and placed into the ambulance helicopter was still presented without any visual obstructions.

Additionally, the live broadcasts include uncensored footage of the cries and moans of the wounded trapped under the rubble, which can be understood as highly psychologically distressing. For example, in Broadcaster-2/Image-14, footage of a crying child under the rubble was aired without any blurring. Similar scenes are frequently seen on both channels. In one case, a shocked and disoriented child is shown sitting in an ambulance, staring around in confusion without responding. In the background, the reassuring voices of healthcare workers can be heard. (Broadcaster-1/Image-6)

#### 3.2.4 Rescue under confluence

Almost all rescue videos show a chaotic rush from the entrance of the earthquake wreckage to the ambulances or vehicles transporting the injured. Warnings of overcrowding are rarely given, and when they are, they seem largely ineffective—at least within the timeframe captured in the visual content. (Broadcaster-2/Image-27) However, it is also noteworthy that confluence rescues were sometimes accompanied by intense chanting and takbirs. Such an atmosphere could be distressing and disorienting for individuals or patients in shock. The visual and discursive content in Broadcaster-2/Image-24 can be described as follows: the rescue team and bystanders place the patient in the ambulance amid loud noise and chanting. [As a viewer, we can say that this scene gives the impression that it could be frightening for the patient.] Subsequent footage shifts the focus to the rescue teams, where certain signs of political affiliation, including religious or nationalist symbolism, become noticeable.

## 4 Discussion

Our analysis reveals significant ethical concerns in media coverage of earthquake rescue operations in Turkey, particularly regarding privacy violations and trauma exposure. The findings indicate systematic patterns of ethical transgressions across both public and private broadcasting, suggesting deeply rooted issues in disaster journalism practices. These patterns manifest in two main categories: normalized discursive presentation and privacy-insensitive visual content, each carrying significant implications for victims, society, and media professionals themselves. The intersection of bioethical principles and media ethics becomes particularly evident in our findings, where traditional medical ethics concepts such as privacy, dignity, and non-maleficence converge with journalistic responsibilities for public information and disaster response (14, 28). This convergence creates an “ethical intersection zone” where medical, humanitarian, and media ethics principles must be carefully balanced.

### 4.1 Normalization of privacy violations and its implications

The most striking finding is the systematic normalization of privacy violations in rescue coverage. Normalizing privacy violations in rescue coverage represents a departure from established medical and disaster ethics principles. The consistent presentation of individuals identities, medical conditions, and moments of extreme vulnerability without apparent ethical consideration by disaster journalism causes the normalization of ethical transgressions in such situations. The normalization of this situation is especially troubling because victims cannot genuinely agree to be exposed in this way, or patients remain silent during their most vulnerable times (3, 29). As some authors have mentioned for journalists, reporting with a reflexive approach that is sensitive to the problems experienced by other people can be an important starting point for avoiding many ethical problems (15).

Our findings regarding multiple cameras at rescue sites, including personal mobile devices, reveal what we term a “privacy violation cascade,” where initial privacy breaches by one media outlet become normalized and encourage further violations. This phenomenon extends beyond traditional media to citizen journalism, corresponding with Vasterman et al.'s (30) observations about media amplification effects in disasters. The presence of this pattern across both public and private broadcasters suggests a systemic issue rather than isolated incidents. It is, therefore, important not only to remember the media's role in following the news but also to be aware of the media's power to determine and influence subsequent social behavior (31). In other words, journalism sensitive to confidentiality and privacy can set an example for other actors, while insensitivity can facilitate insensitive behavior by other actors. Some scholars propose that emotionally charged or individualized disaster coverage can operate as moral witnessing, making human suffering visible and mobilizing empathy and solidarity (15, 32). From this view, certain intense portrayals may honor rather than violate dignity. Our findings acknowledge this potential while suggesting that, without trauma-informed safeguards, moral witnessing can slide into spectacularization, where the quest for emotional resonance eclipses respect for privacy and non-maleficence.

### 4.2 Emotional exploitation and the “rescue show” phenomenon

Our findings about emotional and dramatic presentation styles reveal a concerning trend toward the spectacularization of rescue operations. Using terms like “miracle” and “tearful reunion,” which are documented in our analysis, while perhaps intended to create an emotional connection with viewers, risks transforming human suffering into entertainment. This approach may generate immediate viewer engagement but potentially compromises victims dignity and psychological wellbeing. It is important to note that the irresponsible use of this approach is not in line with the media's responsibility to contribute to the wellbeing and resilience of society (8). It is, therefore, essential to pay close attention to the mental and ethical implications of the language used.

The observed crowds and audible religious or political chanting at rescue sites illustrate the performative component of disaster news as outlined in our research. While potentially sincere, the expressions of community support need to be carefully examined in terms of their impact on the traumatized and the effectiveness of rescue efforts. The presence of the media raises further ethical issues as it can influence the actions of rescue workers and bystanders, potentially compromising both the effectiveness and dignity of rescue efforts (33). It is also significant that this attitude poses a risk to the availability of health services for those rescued and may also affect the working conditions of rescue personnel, raising questions about the balance between public information needs and operational effectiveness. From a bioethical perspective, there is a conflict between the principles of beneficence and non-maleficence (28), where the perceived benefit of public information must be weighed against the potential harm to rescue operations and the dignity of victims (8).

### 4.3 Physical and psychological trauma exposure

The observed violations of medical privacy, including filming inside ambulances and during medical interventions, represent a significant ethical concern extending beyond media ethics into medical professional ethics. This intersection of media coverage and medical care creates competing ethical imperatives between public information rights and individuals rights to privacy and dignified medical care (4). The unrestricted broadcasting of victims suffering moments, including children's distress, violates fundamental principles of medical privacy and human dignity (4). This practice may have long-term implications for victims psychological recovery and raises questions about informed consent in crises. Open displays of physical and psychological trauma raise particular concerns about re-traumatization. Recent research findings indicate that exposure to media content depicting earthquake events can exert a prolonged effect on viewers, with the potential to induce trauma even 3 months after the event (34). This observation contrasts to the conventional understanding of the media's function in promoting recovery and resilience in the aftermath of disasters. Moreover, approximately 2 years after the earthquake, links to television channel content remain readily available at the time of writing this article, highlighting the persistent visibility of such distressing material.

### 4.4 Impact on media professionals and ethical awareness for everyone

A significant implication of our findings concerns the psychological impact on journalists themselves. While our primary analysis focused on content, the documented patterns of intensive emotional coverage suggest potential risks for media professionals. Recent research shows that journalists covering disasters face significant risks of moral injury and PTSD (35). The emotional demands of constant exposure to trauma, combined with the pressure to produce sensational content—evident in our findings of dramatic presentation styles—can lead to significant psychological stress among media professionals (12). Disaster-specific ethical awareness must be reflected in practice to ensure the long-term wellbeing of victims, society, and media personnel. In this respect, it is vital for the media and broadcasting sector, at the institutional level, to ensure a balance between accurate and timely reporting and sensational news or traumatizing, overly emotive reporting. It is imperative for broadcasting organizations to adopt balanced approaches to the presentation of traumatic content in their broadcasts, as this will not only mitigate ethical concerns but also have a favorable impact on the mental wellbeing of journalists.

## 5 Limitations

This study focused exclusively on publicly available television and online broadcasts, without analyzing journalists intentions or institutional decision-making processes. Future work could incorporate interviews with journalists or content-producers and healthcare workers to provide a more comprehensive understanding of the ethical reasoning behind disaster coverage practices. As researchers trained in medical ethics and the history of medicine, our interpretive stance is shaped by disciplinary sensitivity to dignity, privacy, and harm. We acknowledge that this orientation may foreground certain ethical aspects while underrepresenting journalistic rationales. Accordingly, we interpret the results as bioethically informed readings, not definitive judgments of intent.

## 6 Conclusion

This study demonstrates that disaster journalism in Turkey during the 2023 earthquakes exhibited systematic ethical tensions that undermine core bioethical principles of privacy, dignity, and non-maleficence. The systematic nature of privacy violations and emotional exploitation, evident across both public and private broadcasting, suggests these are not isolated incidents but rather normalized practices. Particularly troubling is the routine exposure of victims during their most vulnerable moments, from rescue operations to medical interventions, without apparent consideration for privacy or dignity. The patterns of normalized discursive presentation, characterized by emotional-dramatic framing and planned rescue coverage, point to a concerning transformation of rescue operations into media events. This and the privacy-insensitive visual content showing medical interventions and trauma responses, demonstrate a significant departure from established bioethical principles. The persistence of these broadcasts online, accessible long after the event, raises additional concerns about prolonged trauma exposure for both victims and viewers. These findings suggest an urgent need to reconsider how disaster journalism balances public information needs with ethical responsibilities. Media organizations must develop more robust guidelines for protecting individual dignity during crisis coverage while maintaining their crucial role in disaster response and recovery. The intersection of medical ethics and media responsibilities requires particular attention, especially regarding privacy during medical interventions and trauma exposure. Future research should examine how media organizations can better integrate ethical awareness into urgent reporting situations and evaluate the long-term psychological impacts of privacy violations in disaster coverage. Our study ultimately underscores the critical importance of maintaining ethical standards even in crises, where the pressure for immediate coverage must be balanced against the fundamental human dignity of disaster victims. Integrating trauma-informed ethics into disaster journalism training and institutional codes could mitigate future ethical harms. Strengthened collaboration between bioethicists and media regulators may enhance preparedness for ethically responsible crisis communication.

## Data Availability

The original contributions presented in the study are included in the article/supplementary material, further inquiries can be directed to the corresponding author.
